# Antibiotic susceptibility and genomic variations in *Staphylococcus aureus* associated with Skin and Soft Tissue Infection (SSTI) disease groups

**DOI:** 10.1186/s12879-016-1630-z

**Published:** 2016-06-10

**Authors:** Chih-Hsuan Changchien, Shu-Wun Chen, Ying-Ying Chen, Chishih Chu

**Affiliations:** Department of Plastic and Reconstructive Surgery, Chiayi Christian Hospital, 539 Jhongsiao Rd., Chiayi City, 60002 Taiwan Republic of China; Department of Microbiology, Immunology, and Biopharmaceuticals, National Chiayi University, No 300, University Road, Chiayi, 60004 Taiwan Republic of China

**Keywords:** MRSA, MSSA, SSTI, MLST, Pulsotype

## Abstract

**Background:**

*Staphylococcus aureus* is associated with human skin and soft tissue infections (SSTIs); however, the involvement of virulence factors in different clinical presentations is unclear.

**Methods:**

We analyzed methicillin-resistant *S. aureus* (MRSA) and methicillin-sensitive *S. aureus* (MSSA) strains from Taiwan to determine correlations among the clinical characteristics of SSTIs, antimicrobial susceptibility and virulence factors of *S. aureus* with specific genetic backgrounds.

**Results:**

We identified 177 MRSA isolates and 130 MSSA isolates among the 307 SSTI-associated *S. aureus* isolates. Hospital-acquired (HA)- and community-acquired (CA)-MRSA isolates accounted for 61.6 % and 38.4 % of the isolates, respectively. Clinical presentations in SSTI patients differed significantly for the disease groups. Deep-seated MRSA infections presented with higher amputation rate than MSSA infections. MRSA isolates were all susceptible to linezolid, teicoplanin, and vancomycin, and >94 % of isolates were erythromycin- and clindamycin-resistant. Staphylococcal cassette chromosome (SCC*mec*) types IV, V, and VII were the most frequent in the CA-MRSA group (*n* = 68); types III, IV and V were the most frequent in the HA-MRSA group (*n* = 109). Panton-Valentine leukocidin (PVL) genes were significantly more frequent in CA-MRSA strains (75.0 %) than in HA-MRSA (33.0 %) and MSSA (24.6 %) and were found in 66.7 % (74/111) strains isolated from the abscess group. Exfoliatin A genes were more common in catheter-related exit-site MSSA infections (37.5 %) compared with other MSSA disease groups (*P* < 0.05). Exfoliatin B and superantigen exotoxin genes were uncommon in all SSTI disease types. Pulsotypes A (ST239), C, and D (ST59) were the predominant MRSA genotypes in deep-seated infections.

**Conclusions:**

If not treated appropriately, deep-seated MRSA-associated infections present with higher amputation rates than deep-seated MSSA-associated infections. PVL-positive MRSA strains caused more frequently pus-forming lesions and less bacteremia and invasive diseases. Methods for discriminating CA-MRSA from HA-MRSA strains are now unreliable due to circulation of both ST 239 and ST 59 strains in the community and nosocomial settings. Initial antibiotic treatments should consider MRSA for patients with SSTIs in areas where MRSA is prevalent.

## Background

*Staphylococcus aureus* is a major pathogen responsible for skin and soft tissue infections (SSTIs) in humans. The number of SSTI episodes with a culture-confirmed pathogen increased from 11 % to 24 % between 1998 and 2009, while the incidence of methicillin-resistant *S. aureus* (MRSA) increased from 5 % in 1998 to 42 % in 2005 before decreasing to 37 % in 2009 [1]. In the USA, colonization by *S. aureus* is high among SSTI patients, particularly that of USA300 MRSA [2]. In Taiwan, the prevalence of MRSA in most of the major hospitals ranged from 53 % to 83 % [3]; MRSA is a major pathogen related to SSTIs [4].

It is considered that community-acquired (CA)-MRSA strains are more virulent than hospital-acquired (HA)-MRSA because they possess specific virulence factors and they spread more readily than CA-methicillin-sensitive *S. aureus* (CA-MSSA) [5, 6]. Various extracellular proteins produced by *S. aureus* have been reported to mediate diseases with distinct cutaneous and systemic features. For example, superantigen exotoxin (TSST-1) is associated with staphylococcal toxic shock syndrome, exhibiting desquamative skin rash and multiple organ failure [7]. Exfoliatin A (ETA) and exfoliatin B (ETB) are associated with staphylococcal scalded-skin syndrome with localized and generalized blister formation [8]. Panton-Valentine leukocidin (PVL) and γ-hemolysin (Hlg) target immune cells, such as polymorphonuclear cells and macrophages, to cause pus-forming SSTIs [9]. However, the actual correlations between *S. aureus* virulence factors and the pathogenesis of different SSTI clinical presentations are unclear.

The surgical timing and the choice of empiric antimicrobials when treating *S. aureus*-associated SSTI are still controversial because of different clinical presentations and regional variations in their prevalence [10]. In this preliminary study, the clinical characteristics of SSTIs, antimicrobial susceptibilities, and the virulence factors of *S. aureus* strains with specific genetic backgrounds were investigated.

## Methods

### Patient characteristics

This study was performed at the Chiayi Christian Hospital, a 1000-bed teaching hospital in southern Taiwan, between January 2008 and November 2008 and was approved by the research ethics committee of the Chiayi Christian Hospital (097032 and 101051). The medical records of patients diagnosed with SSTI were reviewed, including demographic data, the type of infection, and underlying chronic illnesses (e.g., diabetes mellitus, hypertension, chronic liver disease, coronary artery disease, chronic renal insufficiency, chronic obstructive pulmonary disease, or malignancy). We categorized 307 cases of *S. aureus* SSTIs on the basis of peculiarity and severity of their clinical presentations into six distinct groups as follows: deep-seated infection (necrotizing fasciitis, osteomyelitis, pyomyositis, and septic arthritis), cutaneous infection (cellulitis, bullous impetigo, furuncle, carbuncle, and staphylococcal scalded-skin syndrome), abscess requiring surgical intervention, postpartum mastitis, catheter-related (peritoneal catheter or intravascular catheter) exit-site infection, or SSTI-related bacteremia. We also documented the microbiological data, appropriateness of empiric antibiotics, number of surgical interventions and reconstructions, need for amputation, length of hospital stay, and the in-hospital mortality rate.

### Bacterial isolates

In total, 307 SSTI-associated *S. aureus* were identified based on their characteristic colony morphology, Gram staining, and the catalase reaction (Staphaurex Plus, Remel, KS, USA). Blood cultures were processed using a VITEK 2 GP system (BioMerieux Vitek, Hazelwood, MO, USA) in the hospital laboratory. *S. aureus* was further confirmed by polymerase chain reaction (PCR) to detect the *clfA*, 16S rRNA, and *nuc* genes, as described previously [11]. BCRC 10781 and BCRC 15211 were used as the methicillin-susceptible and -resistant reference strains, respectively. The HA-MRSA and CA-MRSA isolates were defined as described previously [12].

### Antimicrobial susceptibility testing

Antimicrobial susceptibility was tested using the disc diffusion method according to the guidelines of the Clinical and Laboratory Standards Institute [13]. We determined the susceptibility to clindamycin, erythromycin, fusidic acid, linezolid, oxacillin, penicillin, rifampicin, teicoplanin, tetracycline, trimethoprim-sulfamethoxazole, and vancomycin (BBL, BD, USA). Isolates were considered to be susceptible isolates when their zones of inhibition conformed to the intermediate susceptibility category for a given antibiotic.

### PCR identification of staphylococcal cassette chromosome (SCC*mec*) types and genes for virulence factors

SCC*mec* types I–IV were identified by multiplex PCR amplification of the SCC*mec* region [14, 15]. If isolates could not be assigned to types I–IV, they were grouped into SCC*mec* types V or VII based on the PCR detection of *ccrC* (*ccr5*) homologues [16–18]. Genes for virulence factors, namely PVL, Hlg, TSST-1, ETA, and ETB, were identified by simplex and multiplex PCR amplification using primers, as described previously [19, 20].

### Genetic analysis by pulsed field gel electrophoresis (PFGE) and multilocus sequence typing (MLST)

The genotype of each isolate was identified by *Sma*I-digested PFGE analysis, and the results were visually interpreted as previously described [21, 22]. The MLST types of 16 CA- and HA-MRSA isolates were identified using the method described by Enright [23] and by reference to MLST databases (http://saureus.mlst.net).

### Statistical analysis

Statistical analyses were performed using SPSS 12.0 (SPSS Inc, Chicago, IL, USA). The Student’s *t*-test (two-tailed) was used to compare mean values. The clinical and categorical data for SSTI patients infected with MRSA or MSSA were compared by ANOVA (Duncan’s test). *P* < 0.05 was considered statistically significant.

## Results

### Patient characteristics

Clinical *S. aureus* isolates were collected from 307 SSTI patients, among whom 130 (42.3 %) patients were infected by MSSA and 177 (57.7 %) by MRSA. CA-MRSA and HA-MRSA infections accounted for 68 (38.4 %) and 109 (61.6 %) respectively. Abscess requiring surgical intervention (36.2 %) and cutaneous infection (34.2 %) were the most common clinical features of *S. aureus*-associated SSTI, followed by deep-seated infection (16.9 %), SSTI-related bacteremia (11.4 %), catheter-related exit-site infection (8.1 %), and mastitis (5.2 %). MRSA was more prevalent in all disease groups than MSSA. The most significant predisposing factor was diabetes mellitus (33.6 %), followed by chronic renal diseases (23.5 %) and chronic liver diseases (9.7 %).

Univariate analyses of selected clinical characteristics of SSTI showed significant differences in the clinical presentations of various disease types, excluding the mortality rate. For example, the deep-seated infection group had higher comorbidity and amputation rates than did the abscess group, and the SSTI-related bacteremia group had the highest mean age and duration of hospitalization (Tables 1 and 2). However, most of the clinical characteristics of MRSA-associated SSTI were similar to those of MSSA-associated SSTIs for each infection type (Table 3), except MRSA-associated deep-seated infections presented with a higher amputation rate than did MSSA-associated ones (20.6 % *vs.* 0, *P* < 0.05).

**Table 1:** Differences in the clinical characteristics of MRSA-associated SSTIs among disease groups according to univariate analysis

Clinical characteristics	Deep-seated infection (*n* = 34)	Cutaneous infection (*n* = 54)	Abscess (*n* = 62)	Mastitis (*n* = 11)	SSTI-related bacteremia (*n* = 23)	Catheter-related exit-site infection (*n* = 17)	*P*-value
Mean age (years)	58.4^ab^	50.6^bc^	39.7^cd^	30.7^d^	68.3^a^	60.8^ab^	<0.001
Mean comorbidities	2.2^b^ (Range 0–5)	1.9^b^ (Range 0–6)	0.9^c^ (Range 0–5)	0.1^c^ (Range 0–1)	3.4^a^ (Range 0–7)	3.5^a^ (Range 0–7)	<0.001
Mean operative procedure	1.2^a^ (Range 0–3)	0^c^ (Range 0–1)	1.2^a^ (Range 0–6)	0.1^bc^ (Range 0–1)	0.5^b^ (Range 0–2)	0.1^bc^ (Range 0–2)	<0.001
Reconstructive surgery (%)	4 (11.8)^a^	0 (0)^b^	1 (1.6)^b^	0 (0)^b^	0 (0)^b^	0 (0)^b^	<0.01
Amputation (%)	7 (20.6)^a^	0 (0)^b^	2 (3.2)^b^	0 (0)^b^	3 (13.4)^ab^	0 (0)^b^	<0.001
Mean hospitalization days	16.9^ab^	9.1^b^	9.6^b^	0.5^c^	19.2^a^	13.3^ab^	<0.01
Mortality (%)	0 (0)	3 (5.6)	0 (0)	0 (0)	1 (4.3)	0 (0)	0.248

^ab^Values in a row followed by different letters indicate significant difference between groups; *P* < 0.05

**Table 2:** Differences in the clinical characteristics of MSSA-associated SSTI among disease groups according to univariate analysis

Clinical characteristics	Deep-seated infection (*n* = 18)	Cutaneous infection (*n* = 51)	Abscess (*n* = 49)	Mastitis (*n* = 5)	SSTI-related bacteremia (*n* = 12)	Catheter-related exit-site infection (*n* = 8)	*P*-value
Mean age (years)	53.7^ab^	48.0^ab^	39.5^bc^	27.0^c^	59.9^a^	52.9^ab^	<0.05
Mean comorbidities	2.3^ab^ (Range 0–5)	1.5^bc^ (Range 0–6)	0.8^cd^ (Range 0–4)	0^d^ (Range 0)	3.0^a^ (Range 1–7)	3.1^a^ (Range 0–7)	<0.001
Mean operative procedure	1.6^a^ (Range 0–3)	0.02^e^ (Range 0–1)	1.2^ab^ (Range 1–3)	0.2^de^ (Range 0–1)	0.6^cd^ (Range 0–3)	0.9^bc^ (Range 0–2)	<0.01
Reconstructive surgery (%)	4 (22.2)^a^	0 (0)^b^	2 (4.1)^b^	0 (0)^b^	0 (0)^b^	0 (0)^b^	<0.05
Amputation (%)	0 (0)	0 (0)	0 (0)	0 (0)	0 (0)	0 (0)	NA
Mean hospitalization days	13.2^ab^	6.9^b^	7.7^ab^	4.0^b^	18.3^a^	8.4^ab^	<0.05
Mortality (%)	0 (0)	1 (2.0)	1 (2.0)	0 (0)	0 (0)	0 (0)	0.974

^ab^Values in a row followed by different letters indicate significant difference between groups; *P* < 0.05

**Table 3:** Differences in clinical characteristics between MRSA- and MSSA-associated disease groups according to univariate analysis

Clinical characteristics	Deep-seated infection		Cutaneous infection		Abscess		Mastitis		SSTI-related bacteremia		Catheter-related exit-site infection		*P*-value	
	MRSA (*n* = 34)	MSSA (*n* = 18)	MRSA (*n* = 54)	MSSA (*n* = 51)	MRSA (*n* = 62)	MSSA (*n* = 49)	MRSA (*n* = 11)	MSSA (*n* = 5)	MRSA (*n* = 23)	MSSA (*n* = 12)	MRSA (*n* = 17)	MSSA (*n* = 8)	MRSA	MSSA
Mean age (years)	58.4	53.7	50.6	48.0	39.7	39.5	30.7	27.0	68.3	59.9	60.8	52.9	<0.001	<0.05
Mean comorbidities	2.2	2.3	1.9	1.5	0.9	0.8	0.1	0	3.4	3.0	3.5	3.1	<0.001	<0.001
Mean operative procedure	1.2	1.6	0	0.02	1.2	1.2	0.1	0.2	0.5	0.6	0.1	0.9	<0.001	<0.01
Reconstructive surgery (%)	4 (11.8)	4 (22.2)	0 (0)	0 (0)	1 (1.6)	2 (4.1)	0 (0)	0 (0)	0 (0)	0 (0)	0 (0)	0 (0)	<0.01	<0.05
Amputation (%)	7 (20.6)*	0 (0)	0 (0)	0 (0)	2 (3.2)	0 (0)	0 (0)	0 (0)	3 (13.4)	0 (0)	0 (0)	0 (0)	<0.001	NA
Mean hospitalization days	16.9	13.2	9.1	6.9	9.6	7.7	0.5	4.0	19.2	18.3	13.3	8.4	<0.01	<0.05
Mortality (%)	0 (0)	0 (0)	3 (5.6)	1 (2.0)	0 (0)	1 (2.0)	0 (0)	0 (0)	1 (4.3)	0 (0)	0 (0)	0 (0)	0.248	0.974

*Indicates a significant difference between the MRSA and MSSA groups; *P* < 0.05

Antimicrobial agents were generally used to treat these SSTIs in both MRSA- and MSSA-infected patients (86.4 % *vs.* 78.5 %). Most patients (93 %) with MSSA infections received an effective empiric antimicrobial agent. However, 78.1 % of the patients with MRSA infections received ineffective empiric antimicrobial therapy, where β-lactam antimicrobials were mostly used. Vancomycin was initially prescribed to treat 6.2 % of the MRSA infections and 7.7 % of the MSSA infections.

### SCC*mec* typing and antimicrobial susceptibility testing

The SCC*mec* types of 177 MRSA isolates are shown in Table 4. The *mecA* genes were identified in all (68/68) CA-MRSA strains, 99.1 % (108/109) of HA-MRSA strains, and 2.3 % (3/130) of MSSA strains. The major SCC*mec* types were small-sized SCC*mec* types IV, V, and VII in CA-MRSA strains, and SCC*mec* types III, IV, and V in HA-MRSA strains. SCC*mec* III-carrying isolates were more prevalent in patients with catheter-related exit-site infections (35.3 %) and SSTI-related bacteremia (34.8 %) than patients with mastitis (9.0 %) or abscesses (8.1 %) (*P* < 0.05). SCC*mec* VII-carrying isolates were more prevalent in patients with abscesses (27.4 %) than in those with catheter-related exit-site infection (0) (*P* < 0.05) (Table 5).

**Table 4:** Differences in prevalence of SCC*mec* types and virulence factors among CA-MRSA, HA-MRSA, and MSSA groups

SCC*mec* types and virulence factors	CA-MRSA	HA-MRSA	MSSA	*P*-value
	(*n* = 68)	(*n* = 109)	(*n* = 130)	
SCC*mec*				
Type II	1 (1.4 %)^c^	4 (3.6 %)^b^	0 (0)	0.393
Type III	4 (5.9 %)^c^	27 (24.8 %)^a^	0 (0)	<0.01
Type IV	20 (29.4 %)^a^	25 (22.9 %)^a^	0 (0)	0.339
Type V	21 (30.9 %)^a^	29 (26.6 %)^a^	0 (0)	0.541
Type VII	15 (22.1 %)^ab^	14 (12.8 %)^b^	0 (0)	0.108
Nontypeable	7 (10.3 %)^bc,y^	10 (9.2 %)^b,y^	130 (100 %)^x^	<0.01
*mecA*	68 (100 %)^a^	108 (99.1 %)^a^	3 (2.3 %)^b^	<0.01
Virulence factors				
PVL	51 (75.0 %)^x^	36 (33.0 %)^y^	32 (24.6 %)^y^	<0.01
Hlg	68 (100 %)^x^	109 (100 %)^x^	120 (92.3 %)^y^	<0.01
TSST-1	4 (5.8 %)	4 (3.7 %)	7 (4.6 %)	0.725
ETA	14 (22.1 %)^y^	35 (29.6 %)^x^	23 (19.0 %)^y^	<0.01
ETB	1 (1.4 %)	0 (0)	0 (0)	0.09

^ab^Different letters in the same column indicate significant differences between groups; *P* < 0.05

^xy^Different letters in the same row indicate significant differences between groups; *P* < 0.05

**Table 5:** Differences in the prevalence of SCC*mec* types and virulence factors of MRSA among disease groups

SCC*mec* type	Deep-seated infection (*n* = 34)	Cutaneous infection (*n* = 54)	Abscess (*n* = 62)	Mastitis (*n* = 11)	SSTI-related bacteremia (*n* = 23)	Catheter-related exit-site infection (*n* = 17)	*P*-value
Type II	2 (5.9 %)	2 (3.7 %)	1 (1.6 %)	0 (0)	0 (0)	0 (0)	0.608
Type III	9 (26.5 %)^ab^	10 (18.5 %)^ab^	5 (8.1 %)^b^	1 (9.0 %)^b^	8 (34.8 %)^a^	6 (35.3 %)^a^	<0.05
Type IV	9 (26.5 %)	14 (25.9 %)	14 (22.6 %)	4 (36.4 %)	6 (26.1 %)	4 (23.5 %)	0.936
Type V	7 (20.5 %)	17 (31.5 %)	15 (24.2 %)	5 (45.5 %)	4 (17.4 %)	6 (35.3 %)	0.294
Type VII	4 (11.8 %)^ab^	8 (14.8 %)^ab^	17 (27.4 %)^a^	1 (9.0 %)^ab^	3 (13.0 %)^ab^	0 (0)^b^	<0.05
Non-typeable	3 (8.8 %)	3 (5.0 %)	10 (16.1 %)	0 (0)	2 (8.7 %)	1 (5.9 %)	0.101

^ab^Values in a row followed by different letters indicate significant differences between groups; *P* < 0.05

**Fig. 1:**
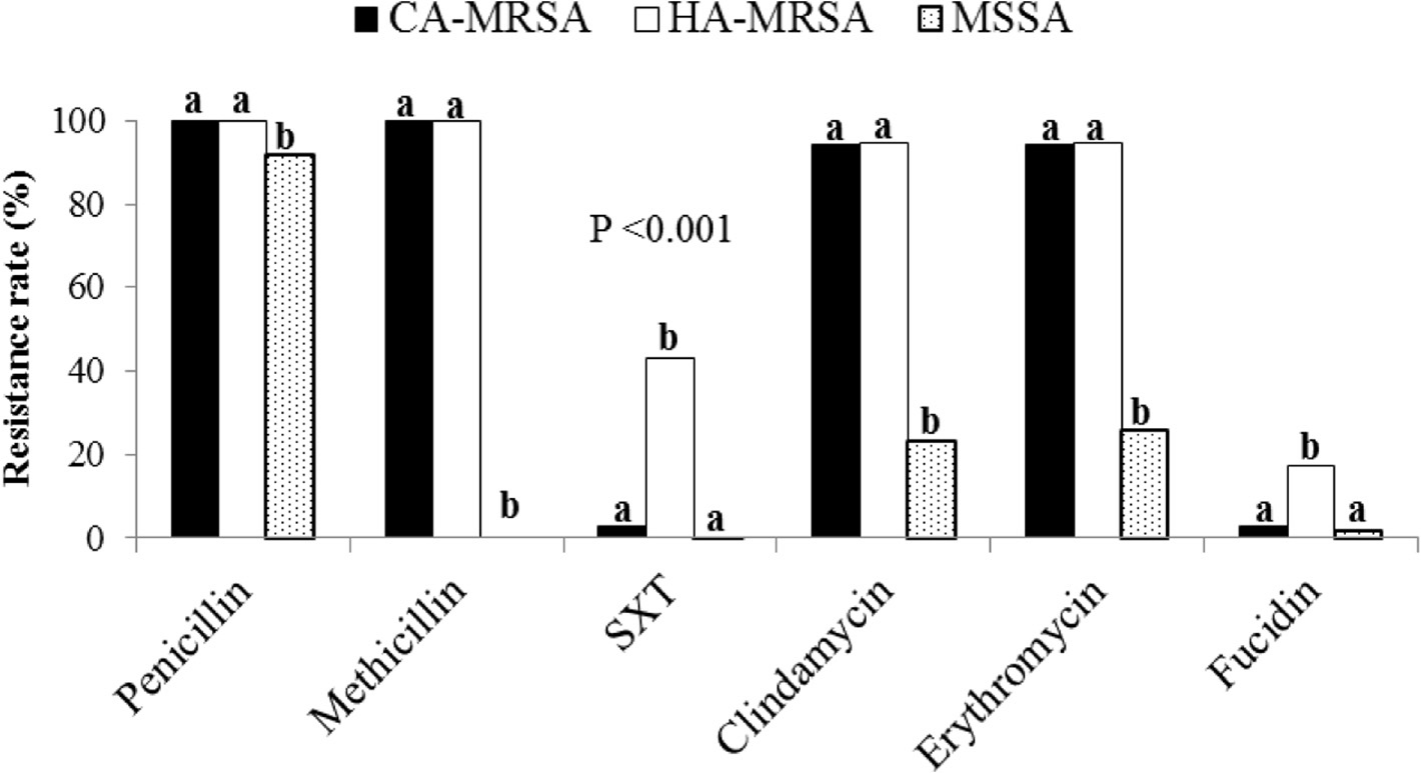
Antimicrobial susceptibility of CA-MRSA, HA-MRSA, and MSSA. Different letters (a and b) indicate significant differences between groups; *P* < 0.05

The antibiotic resistance patterns differed significantly among the various SCC*mec* types. The majority of the isolates that contained SCC*mec* IV, V, or VII elements were susceptible to trimethoprim-sulfamethoxazole (SXT) but were resistant to β-lactams, erythromycin, and clindamycin. SCC*mec* type II or III isolates were more resistant to fusidic acid than SCC*mec* type IV, V, or VII isolates (*P* < 0.05). All *S. aureus* isolates were susceptible to linezolid, teicoplanin, and vancomycin. Most MSSA isolates were susceptible to all antibiotics tested, excluding penicillin. Over 94 % MRSA isolates were resistant to erythromycin and clindamycin. Compared with CA-MRSA isolates, HA-MRSA isolates were more resistant to fusidic acid and SXT (Fig. 1).

### Prevalence of PVL, Hlg, TSST-1, ETA, and ETB genes

The PVL gene was identified in 38.8 % *S. aureus* isolates and was significantly more frequent in CA-MRSA strains (75.0 %, 51/68) than in HA-MRSA (33.0 %, 36/109) and MSSA strains (24.6 %, 32/130) (*P* < 0.01) (Table 4). Irrespective of the methicillin-resistance phenotype, PVL genes were detected most frequently in abscesses, i.e., 66.7 % (74/111), followed by mastitis, cutaneous infections, deep-seated infections, bacteremia, and catheter-related infections. Among all of the SSTI cases, only one ETB gene was identified from a patient with staphylococcal scalded-skin syndrome. Compared with other MSSA disease groups, ETA genes were more common in catheter-related exit-site MSSA infections (37.5 %) (*P* < 0.05). All the MRSA isolates possessed Hlg genes, which were otherwise only present in 92.3 % of the MSSA isolates (*P* < 0.01). The TSST-1 gene was seldom identified in any disease type (Table 6).

**Table 6:** Differences in virulence factors between MRSA and MSSA among disease groups

Virulence factor	Deep-seated infection		Cutaneous infection		Abscess		Mastitis		SSTI-related bacteremia		Catheter-related exit-site infection	
	MRSA (*n* = 34)	MSSA (*n* = 18)	MRSA (*n* = 54)	MSSA (*n* = 51)	MRSA (*n* = 62)	MSSA (*n* = 49)	MRSA (*n* = 11)	MSSA (*n* = 5)	MRSA (*n* = 23)	MSSA (*n* = 12)	MRSA (*n* = 17)	MSSA (*n* = 8)
PVL	7 (20.6 %)	1 (5.6 %)	27 (50.0 %)*	4 (7.8 %)	49 (79.0 %)	25 (51.0 %)	5 (45.5 %)	2 (40.0 %)	3 (13.0 %)	0 (0)	1 (5.9 %)	0 (0)
Hlg	34 (100 %)	17 (94.4 %)	54 (100 %)	47 (92.2 %)	62 (100 %)	46 (93.9 %)	11 (100 %)	4 (80.0 %)	23 (100 %)	12 (100 %)	17 (100 %)	7 (87.5 %)
TSST-1	4 (11.8 %)	0 (0)	2 (3.7 %)	2 (3.9 %)	2 (3.2 %)	5 (10.2 %)	0 (0)	0 (0)	1 (4.3 %)	1 (8.3 %)	0 (0)	1 (12.5 %)
ETA	7 (20.6 %)	4 (22.2 %)	15 (27.8 %)	12 (23.5 %)	18 (29.6 %)	2 (4.1 %)	3 (27.3 %)	1 (20.0 %)	5 (21.7 %)	3 (25.0 %)	5 (29.4 %)	3 (37.5 %)
ETB	0 (0)	0 (0)	1 (1.9 %)	0 (0)	0 (0)	0 (0)	0 (0)	0 (0)	0 (0)	0 (0)	0 (0)	0 (0)

*Indicates a significant difference between MRSA and MSSA groups; *P* < 0.05

### PFGE analysis

PFGE analysis separated 32 MRSA isolates from deep-seated infections into eight pulsotypes, among which pulsotypes A, C, and D accounted for 78.1 % of the isolates. Associations between pulsotypes and SCC*mec* types were determined for pulsotype A with SCC*mec* type II

I (100 %) and pulsotype C with SCC*mec* type IV (88.9 %). Pulsotype D contained diverse SCC*mec* elements (types II, III, V, VII, and nontypeable SCC*mec* elements), but SCC*mec* type V was the major type (40 % of pulsotype D). MLST analysis identified pulsotype A as ST239 and pulsotype C as ST59.

## Discussion

*S. aureus*-associated SSTIs exhibit diverse clinical presentations, mediated by various bacterial toxins and host susceptibility to some extent. Localized cutaneous infections in healthy individuals may resolve spontaneously using topical antimicrobials. However, the majority of abscess-forming lesions usually require surgical intervention. Aggressive antimicrobial treatment of deep-seated infections and SSTI-related bacteremia is crucial for survival. Compared with MSSA infections, MRSA infections may present with different clinical characteristics and outcomes for deep-seated infections, bacteremia, catheter-related infections, and mastitis [24–26]. It has been reported that patients with MRSA bacteremia have a significantly higher mortality rate (15–60 %) and longer hospitalization duration compared with those with MSSA bacteremia [27]. By contrast, Miller *et al*. reported that most clinical characteristics of MRSA-associated SSTI could not be used as markers to distinguish MSSA-infected patients [28]. Our results indicate that deep-seated MRSA infections presented with a higher amputation rate than MSSA infections (20.6 % *vs.* 0 %) (Table 3). It should be noted that most patients (78.1 %) with MRSA infections received ineffective empiric antimicrobial therapy. Thus, the correlation between diverse clinical outcomes and inappropriate empiric therapy for severe infections requires further study.

Catheter-related exit-site infection is a peculiar nosocomial infection, which is usually associated with bacterial colonization and biofilm formation in SSTIs, and has been reported as the most common source of *S. aureus* bacteremia, with equal proportions of MSSA and MRSA infection [29]. Here, patients with catheter-related exit-site MRSA infections were more prevalent and had more concurrent bacteremia (64.7 % *vs.* 0) compared with MSSA infections, suggesting a greater prevalence of MRSA in our hospital.

In general, *S. aureus*, β-hemolytic *streptococcus*, *Escherichia coli*, and *Bacteroides spp.* have been isolated from patients with postpartum mastitis. Among the mastitis-associated MRSA isolates obtained in this investigation, 10/11 (90.9 %) were classified as HA-MRSA strains, but their antimicrobial resistance patterns and PCR results indicated that most isolates possessed the characteristics of CA-MRSA strains.

According to the isolates analyzed by PFGE, the predominant MRSA genotypes were pulsotypes A, C, and D. Pulsotype A isolates related to ST 239 are the major HA-MRSA clones in Taiwan [30], whereas pulsotype C and D isolates related to ST 59 are the prevalent clones of CA-MRSA in Taiwan [3, 31]. However, we identified both ST 239 and ST 59 strains from CA and HA-SSTI patients, thereby implying that both strains are circulating concomitantly in community and nosocomial settings.

It was notable that there was a significant association between PVL and MRSA, particularly in cutaneous abscess compared with bacteremia and invasive illnesses. These findings suggest that PVL-positive strains have a higher tendency to develop pus-forming lesions that often require surgical drainages. PVL-positive isolates were significantly more prevalent among the CA-MRSA strains compared with MSSA strains, as described previously [32], thereby reflecting the consequences of the widespread use of β-lactam antibiotics, leading to the selection of a few restricted PVL-positive MRSA strains. Furthermore, PVL genes were identified in both CA- and HA-MRSA strains that possessed SCC*mec* type III, although SCC*mec* type IV isolates were dominant in both the CA- and HA-MRSA groups (Table 4). Our results suggest that the SCC*mec* types and the presence of PVL genes are not useful molecular markers for distinguishing between CA- and HA-MRSA strains.

TSST-1, ETA, and ETB are reportedly the major virulence factors associated with some peculiar skin and systemic syndromes in clinical settings. The low carriage rate of these genes in *S. aureus* isolates from SSTI patients could explain the low incidence of staphylococcal toxic shock syndrome and staphylococcal scalded-skin syndrome in the present study. Diverse skin and systemic presentations of various disease types of SSTI may be correlated with other exotoxin genes or combinations of virulence factors. The *mecA* gene was not found in an MRSA isolate in this study, which could be related to diverse resistance mechanisms, e.g., the production of β-lactamase or other unidentified factors that may contribute to resistance in MRSA [33]. Furthermore, three MSSA isolates that possessed the *mecA* gene may have been derived from a *mecA*-positive MRSA strain subpopulation with a heterogeneous resistance phenotype that cannot be detected by traditional susceptibility testing [34].

The treatment of SSTIs involves topical agents, surgical intervention, or oral or parenteral antimicrobial therapy. Recent reports and the present study demonstrate the high impact of MRSA in SSTIs [35]. Uncomplicated skin abscesses are curable by surgical drainage alone; antimicrobials are not necessary according to the current guidelines. In areas with a high prevalence of MRSA infections, empiric antimicrobial treatment should consider the possibility of MRSA infection in complicated SSTIs and immunocompromised individuals. CA-MRSA strains with type IV SCC*mec* elements are more susceptible to a variety of non-β-lactam antimicrobials than are HA-MRSA strains [36]; antimicrobial susceptibility patterns have been used to discriminate CA-MRSA and HA-MRSA strains. However, this approach is now unreliable because CA-MRSA may also acquire resistance to non-β-lactam antibiotics [37]. Clindamycin has been used as an option for staphylococcal SSTIs due to its good distribution in skin and wound exudates [38]. However, we found that erythromycin and clindamycin resistance were much more frequent (over 94 %) in both CA- and HA-MRSA isolates than those reported recently from different countries [39]. The treatment guidelines for MRSA infections should be adjusted according to the currently defined efficacy of non-β-lactam antimicrobials. For complicated SSTIs, the treatment of patients with appropriate initial antimicrobials is associated with a statistically significant impact on the cure rate [40]. According to the susceptibility tests in the present study, SXT appears to be an appropriate empiric oral antimicrobial for treating CA-MRSA infections, whereas fusidic acid is more suitable for treating both CA- and HA-MRSA infections (Fig. 1). Vancomycin is warranted only for empiric use when treating deep-seated, life-threatening MRSA infections.

## Conclusions

MRSA is an important emerging pathogen in SSTIs. Most of the clinical characteristics of MRSA-associated SSTIs are similar to those of MSSA-associated SSTIs, except for the higher amputation rate in deep-seated MRSA infections. We found that PVL-positive strains had a higher tendency to cause pus-forming lesions, whereas they were rarely associated with bacteremia and invasive diseases. ETB and TSST-1 genes were identified only rarely in various SSTI disease types. The previously employed criteria and antimicrobial susceptibility patterns for discriminating CA- and HA-MRSA strains are now unreliable because both ST 239 (major clone of HA-MRSA) and ST 59 (major clone of CA-MRSA) strains are circulating concomitantly in community and nosocomial settings in Taiwan. Early surgical drainage of pus-forming lesions and appropriate antimicrobials for invasive infections are mandatory for SSTI treatment. If indicated, the initial empiric antimicrobial treatment should consider MRSA for patients with SSTIs in areas where MRSA is prevalent.

## Abbreviations

CA-MRSA, community-acquired methicillin-resistant *Staphylococcus aureus*; CC, clindamycin; CIP, ciprofloxacin; CLSI, Clinical and Laboratory Standards Institute; ETA, exfoliatin A; ETB, exfoliatin B; ERY, erythromycin; FA, fusidic acid; GM, gentamicin; HA-MRSA, hospital-acquired MRSA; Hlg, γ-hemolysin; LZD, linezolid; MLST, multilocus sequence typing; MSSA, methicillin-susceptible *Staphylococcus aureus*; P, penicillin; PFGE, pulsed field gel electrophoresis; PVL, Panton-Valentine leukocidin; RA, rifampicin; SCC*mec*, Staphylococcal Cassette Chromosome *mec*; SSTI, skin and soft tissue infection; SXT, trimethoprim-sulfamethoxazole; TE, tetracycline; TEC, teicoplanin
